# Clinically relevant circulating microRNA profiling studies in pancreatic cancer using meta-analysis

**DOI:** 10.18632/oncotarget.15148

**Published:** 2017-02-07

**Authors:** Zenglin Pei, Song-Mei Liu, Jing-Tao Huang, Xuan Zhang, Dong Yan, Qianlin Xia, Chunxia Ji, Weiping Chen, Xiaoyan Zhang, Jianqing Xu, Jin Wang

**Affiliations:** ^1^ Scientific Research Center, Shanghai Public Health Clinical Center, Fudan University, Jinshan District, Shanghai 201508, P.R. China; ^2^ Center for Gene Diagnosis, Zhongnan Hospital of Wuhan University, Wuhan, Hubei 430071, P.R. China; ^3^ Department of Medical Oncology, Beijing Chaoyang Hospital affiliated to Capital Medical University, Beijing, China; ^4^ Genomics Core, National Institute of Diabetes and Digestive and Kidney Diseases, National Institutes of Health, Bethesda, MD 20892, USA

**Keywords:** pancreatic cancer, meta-analysis, diagnostics, multiple miRNA, SROC

## Abstract

**Background:**

Pancreatic cancer (PaCa) is the most lethal gastrointestinal (GI) tumor. Although many studies on differentially expressed miRNAs as candidate biomarkers of pancreatic cancer have been published, reliability of these findings generated from investigations performed in single laboratory settings remain unclear.

**Results:**

There were 29 articles with a total of 2,225 patients and 1,618 controls included in this meta-analysis. The pooled sensitivity was 82% (95% CI, 79–85%); the specificity was 85% (95% CI, 79–89%); and area under the curve (AUC) was 0.89 (95% CI, 0.86–0.92). Subgroup analyses indicated that there were significant divergences between Caucasian and Asian subgroups for circulating miRNA analysis.

**Materials And Methods:**

To comprehensively investigate the potential utility of miRNAs as biomarkers of the disease, we searched publications diagnosing PaCa using miRNAs from PubMed, Medline, Embase, Google Scholar and Chinese National Knowledge Infrastructure (CNKI) databases. The sensitivity (SEN), specificity (SPE), and summary receiver operating characteristic (SROC) curve were used to examine the overall test performance, and heterogeneity was analyzed with the *I*^2^ test.

**Conclusions:**

Our analysis demonstrated that multiple miRNAs (SEN: 85%; SPE: 89%; AUC: 0.93) were more accurate for diagnosing PaCa than a single miRNA (SEN: 78%; SPE: 79%; AUC: 0.84), and future studies are still needed to confirm the diagnostic value of these pooled miRNAs for PaCa.

## INTRODUCTION

Pancreatic cancer (PaCa) is one of the most lethal and aggressive cancers, with most patients dying within one year after diagnosis and a less than 6% 5-year survival rate [1]. However, the 5-year survival rate for pancreatic cancer patients increases significantly with curative resection of early-stage disease [2]. K-ras, p53, serum CA19-9 and CEA have been the most widely used biomarkers for PaCa diagnosis [3, 4], but these biomarkers often lead to incorrect diagnosis for PaCa and other non-cancer pancreatic diseases (e.g., chronic pancreatitis) because of their unreliable sensitivity (SEN) and improper specificity (SPE) [4, 5], so that diagnosis of PaCa remains a major clinical challenge. There is therefore an urgent need to identify sensitive and specific biomarkers for early detection of pancreatic cancer. Thus, finding valid, reliable biomarkers for early detection and developing an objective molecular test for PaCa diagnosis will have clear clinical significance.

miRNA is a class of functional double stranded 18–24 nucleotide non-coding RNA molecules that decrease gene expression through translational inhibition or degeneration of target mRNA [6]. Tumor-associated miRNAs activate critical cancer relevant pathways and play key roles in the oncogenic process and are confirmed to be involved in the genetic networks regulating functional pathways in pancreatic cancer, which can be candidate biomarkers of PaCa [7, 8]. Although their diagnostic accuracy has been evaluated and several studies have obtained promising results, the possible application of miRNAs for diagnosing PaCa remains controversial due to wide-ranging values of SEN and SPE in these studies, which may lead to different results dependent on subjects’ ethnicities, sources of controls, types of miRNAs, and specimen. Relatively low diagnostic accuracy was found in studies applying single-miRNA profiling for the diagnosis of PaCa. For example, Zhao et al. investigated the value of miR-192 for the diagnosis of PaCa with an SEN of 76.0% and an SPE of 55.0% in an Asian population [9]. Carlsen et al. investigated the value of miR-375 for the diagnosis of pancreatic ductal adenocarcinoma (PDAC) with SEN of 77.0% and SPE of 66.0% [10], which revealed that in the plasma-miRNA population, miR-375 was increased in PDAC cases compared with patients with other pancreatic or gastrointestinal diseases. The diagnostic accuracy of miR-21 for PaCa was confirmed in another Asian population, with results exhibiting an SEN of 77.8% and an SPE of 66.7%, respectively [11]. Habbe et al. showed an SEN of 81.0% and an SPE of 98.0% in a Caucasian population for the diagnostic accuracy of miR-21 [12]. Moreover, Cote et al. investigated a set of miRNAs (miR-10b, miR-155, miR-106b, miR-30c, and miR-212) as biomarkers for the early diagnosis of PaCa with 95.0% SEN and 100.0% SPE [13]. We were motivated by these discordant results, which were generated from investigations performed in single laboratory settings with minimal evidence of reproducibility and independent validation in other laboratories [14], to conduct a meta-analysis to develop the diagnostic accuracy of miRNA assays for PaCa diagnosis.

## RESULTS

### Systematic review and quality assessment of diagnostic studies of pancreatic cancer for meta-analysis

**Figure 1:**
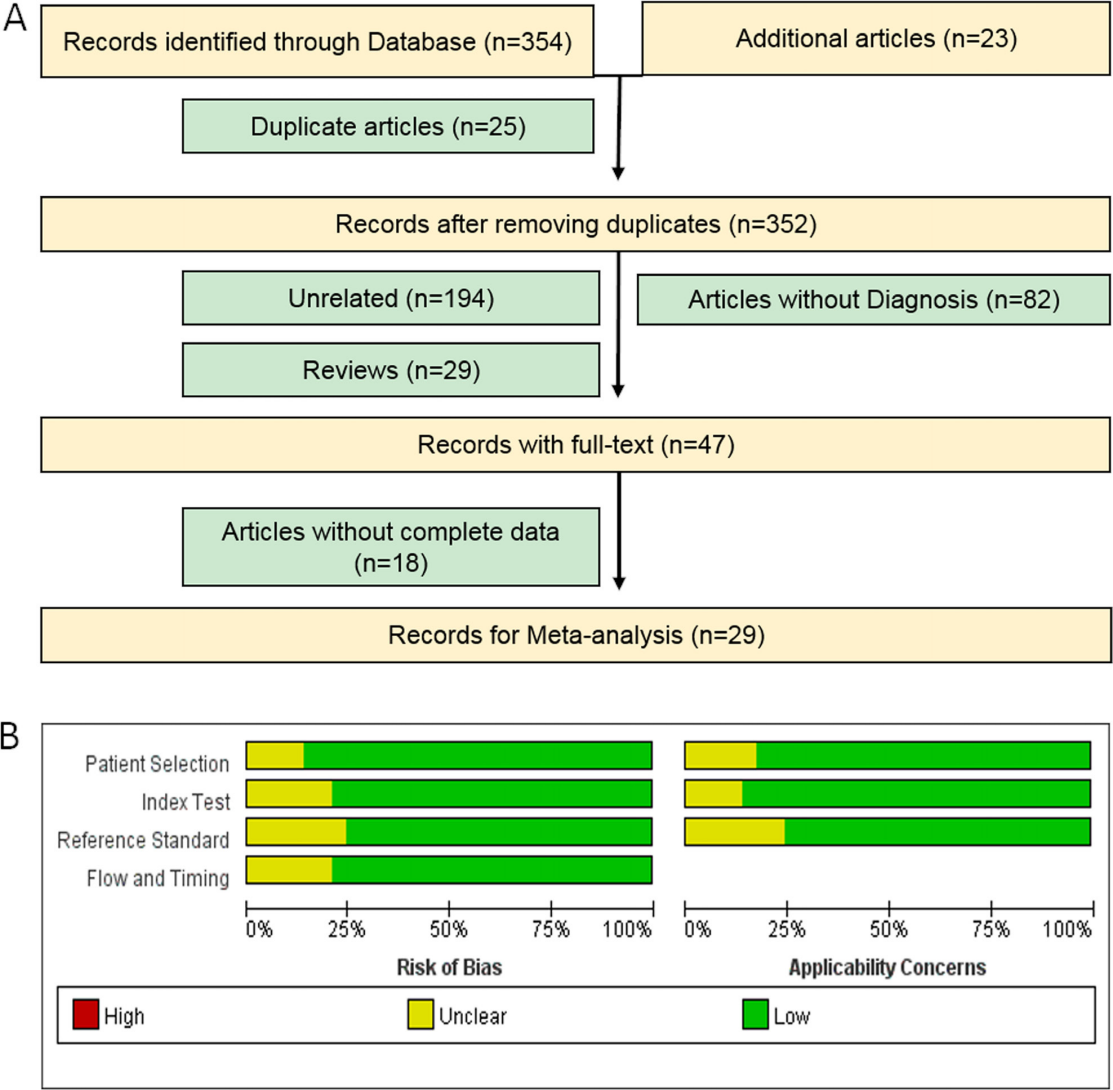
Flow chart of the meta-analysis of miRNA in PaCa (**A**) and quality of included studies according to QUADAS-2 guidelines: proportion of studies with risk of bias; proportion of studies with regarding applicability (**B**).

Based on our primary literature research from PubMed, Medline, Embase, Google Scholar and Chinese National Knowledge Infrastructure (CNKI) databases, there are a total of 354 eligible relevant studies diagnosing PaCa using miRNAs in patients and an additional 23 eligible studies found by scanning the reference lists in our initial study, of which 25 studies were removed as duplicate records (Figure 1A). After screening the titles, abstracts and keywords, we further excluded 294 studies as reviews (*n* = 29), for the study not including miRNAs (*n* = 194), for the study not including PaCa diagnosis (*n* = 82) and lacking complete data (*n* = 18). Ultimately, there are 29 articles with 36 studies [2, 7–13, 15–35] published between 2009 and 2016 examining the efficacy of miRNAs for diagnosing PaCa compared with healthy controls and included a total of 3843 participants (2225 patients with PaCa and 1618 controls) from the United States, Japan, Germany, France, Denmark, and China, shown in Table 1. Habbe et al.'s article [12], Que et al.'s article [23], Cote et al.'s article [13], Xie et al.'s article [33], Humeau et al.'s article [7], Cao et al.'s article [2] and Xu et al.'s article [35] included 2 studies, and the remaining 21 articles included 1 study each [8–11, 15–22, 24–32, 34]. Next, we found that 21 studies were performed in Asian populations and the other 15 studies were performed in Caucasian populations. A total of 25 studies detected miRNA in blood (such as whole blood [24, 26, 28], serum [9, 20, 22, 23, 27, 29, 32, 34] and plasma samples [2, 8, 10, 11, 13, 15, 16, 18, 19, 25, 35]), and 11 studies detected miRNA in non-blood samples (including bile [13], cyst fluid [17], pancreatic juice [12, 30], salivary [7, 33], and stool [21, 31]). We evaluated 19 studies for assessing the diagnostic efficacy of multiple miRNAs [2, 13, 15, 17–22, 26–32, 34] and single miRNAs using meta-analysis for discriminating patients with PaCa from healthy controls in 17 studies [7–12, 16, 23–25, 33, 35] in these 29 articles. The quantitative real-time polymerase chain reaction (qRT-PCR) or immunohistochemistry (IHC) assay were used in these studies for measuring the expression levels of these miRNAs, and the reference miRNAs (Table 1) was used as the endogenous control for normalization, such as RNU6B, RNU44, RNU48, miR-16, miR-24, miR-39, miR-54, miR-238, miR-425-5p, and miR-3196. Moreover, we found that RNU6B, miR-16, and miR-39 were often used as reference miRNAs for miRNAs based studies in pancreatic cancer. The qualities of the selected studies all turned out to be high according to QUADAS-2 guidelines (Figure 1B).

**Table 1:** The main features of included studies in meta-analysis

Reference	Ethnicity	Specimen	PCa		Control		Patient spectrum	miRNA	Reference miRNA	Sensitivity (%)	Specificity (%)
			Number	Age	Number	Age					
Habbe et al. 2009	Caucasian	Pancreatic juice	64	n.a.	54	n.a.	Pancreatic cancer	miR-155	RNU6B	83	93
								miR-21		81	98
Wang et al. 2009	Caucasian	Plasma	28	n.a.	19	n.a.	PDAC	miR-21, miR-210, miR-155, and miR-196a	miR-16	64	89
Liu et al. 2011	Asian	Plasma	45	n.a.	30	n.a.	PDAC	miR-21	miR-39	77.8	66.7
Morimura et al. 2011	Asian	Plasma	36	68	30	58	Pancreatic cancer	miR-18a	RNU6B	90	75
Ryu et al. 2011	Caucasian	Pancreatic cyst fluid	24	57	16	58	Pancreatic cancer	miR-21, miR-221, miR-17-3p	RNU6B	80	76
Bauer et al. 2012	Caucasian	Plasma	45	n.a.	33	n.a.	PDAC	a set of 100 miRNAs	RNU6B	100	93.7
Liu et al. 2012	Asian	Plasma	138	62	175	64	Pancreatic cancer	miR-16, miR-21, miR-155, miR-181a, miR-181b, miR-196a and miR-210	miR-39	64.5	78.9
Liu et al. 2012	Asian	Serum	95	63	81	58	Pancreatic cancer	miR-20a, miR-21, miR-24, miR-25, miR-99a, miR-185 and miR-191	-	89	100
Ren et al. 2012	Asian	Stool	29	63	13	58	Pancreatic cancer	miR-210, miR-196a, miR-181b	miR-16	84.6	69.2
Kawaguchi et al 2013	Asian	Plasma	47	n.a.	30	n.a.	Pancreatic cancer	miR-221	RNU6B	74	78
Li et al. 2013	Caucasian	Serum	41	65	19	44	PDAC	miR-1290 and miR-146a	miR-16	88	84
Wang et al. 2013	Asian	Blood	129	63	163	44	Pancreatic cancer	miR-27a-3p	RNU6B	82.2	76.7
Que et al. 2013	Asian	Serum	22	65	27	58	PDAC	miR-21	RNU6B	95.5	81.5
								miR-17-5p		72.7	92.6
Zhao et al. 2013	Asian	Serum	70	60	40	60	PDAC	miR-192	RNU6B	76	55
Carlsen et al. 2013	Caucasian	Plasma	47	65.5	45	59.5	PDAC	miR-375	RNU6B, miR-238, miR-54	77	66
Ganepola et al. 2014	Caucasian	Blood	11	68	11	46	Pancreatic cancer	miR-642b, miR-885-5p, miR-22-3p	miR-3196	91	91
Schultz et al. 2014	Caucasian	Blood	180	65	199	53	Pancreatic cancer	miR-26b, miR-34a, miR-122, miR-126, miR-145, miR-150, miR-223, miR-505, miR-636 and miR-885.5p	RNU44, RNU48	85	85
Zhang et al. 2014	Asian	Serum	70	n.a.	40	n.a.	PDAC	miR-192 and miR-194	RNU6B	84	75
Slater et al. 2014	Caucasian	Serum	9	n.a.	10	n.a.	PDAC	miR-196a, miR-196b	miR-24	89	90
Wang et al. 2014	Caucasian	Pancreatic juice	50	n.a.	38	n.a.	PDAC	miR-205, miR-210, miR-492, miR-1427	RNU6B	87	88
Cote et al. 2014	Caucasian	Plasma	40	67	25	66	PDAC	miR-10b, miR-155, miR-106b, miR-30c and miR-212	miR-425-5p	95	100
		Bile	40	67	25	66	PDAC	miR-10b, miR-155, miR-106b, miR-30c and miR-212		96	100
Chen et al. 2014	Asian	Plasma	109	n.a.	50	n.a.	Pancreatic cancer	miR-182	RNU6B	64.1	82.6
Lin et al. 2014	Asian	Serum	49	62	27	61	Pancreatic cancer	miR-492, miR-663a	miR-39	75.5	70
Yang et al. 2014	Asian	Stool	30	n.a.	25	n.a.	PDAC	miR-21, miR-155, miR-216	RNU6B	83.33	83.33
Xie et al. 2015	Asian	Salivary	40	n.a.	40	n.a.	Pancreatic cancer	miR-3679-5p	RNU6B	82.5	45
								miR-940		90	40
Humeau et al 2015	Caucasian	Salivary	7	67	4	70	PDAC	miR-21	-	71.4	100
								miR-23		85.7	100
Kojima et al 2015	Asian	Serum	100	n.a.	21	n.a.	Pancreatic cancer	miR-6075, miR-4294, miR-6880-5p, miR-6799-5p, miR-125a-3p, miR-4530, miR-6836-3p and miR-4476	-	80.3	97.6
Cao et al. 2016	Asian	Plasma	156	n.a.	57	n.a.	Pancreatic cancer	miR-486-5p, miR-126-3p, miR-106b-3p	RNU6B	82.7	84.4
		Plasma	29	n.a.	16	n.a.	Pancreatic cancer	miR-486-5p, miR-126-3p, miR-106b-3p, miR-938, miR-26b-3p, and miR-1285		83.9	80.8
Xu et al 2016	Asian	Plasma	156	n.a.	65	n.a.	Pancreatic cancer	miR-938	RNU6B	61.5	73.8
								miR-486-5p		75	87.7

### Sensitivity and specificity of circulating miRNAs for the diagnosis of pancreatic cancer

**Figure 2:**
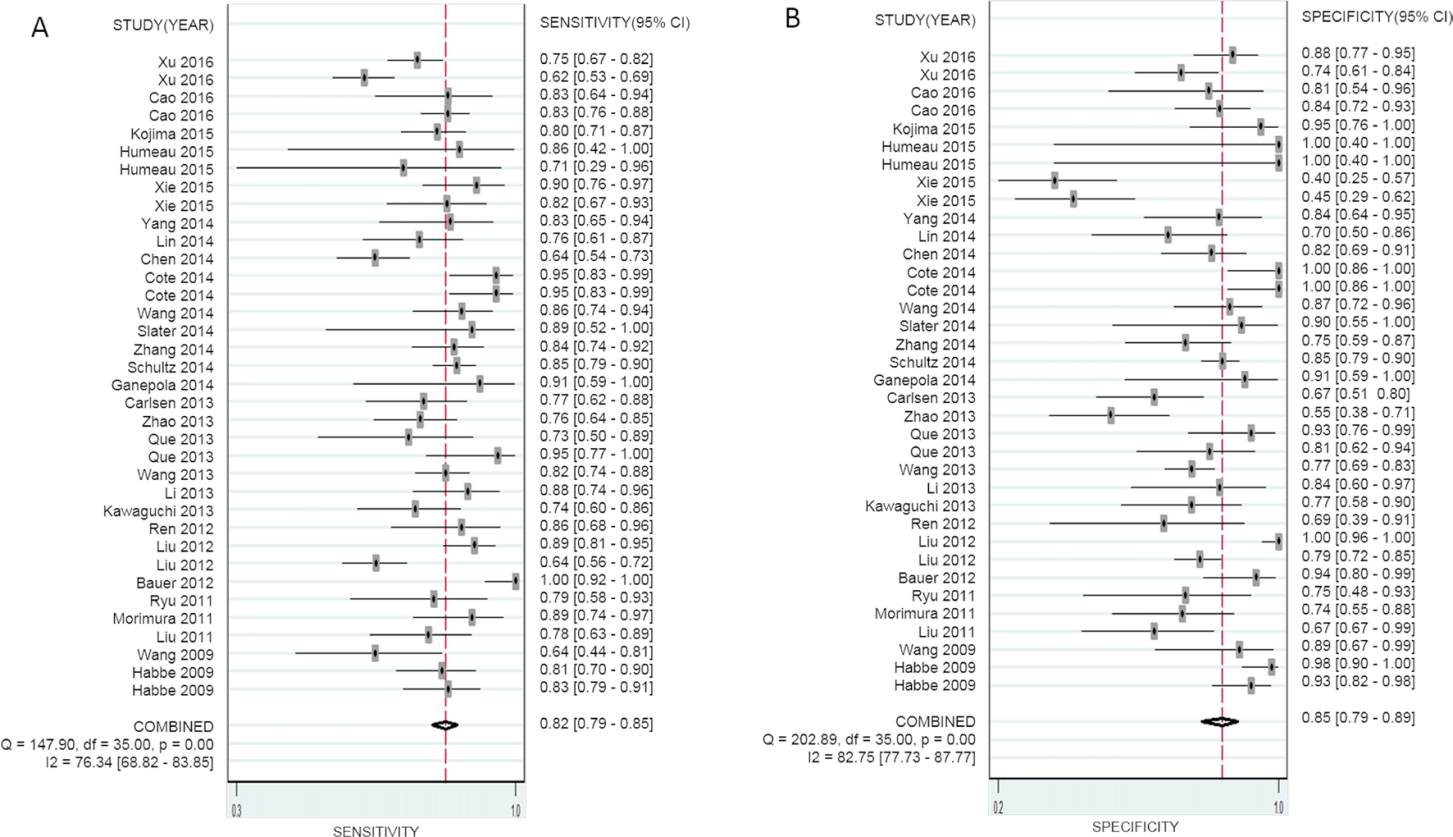
Forest plots of sensitivity (**A**) and specificity (**B**) with corresponding heterogeneity statistics for miRNA in the diagnosis of pancreatic cancer.

**Figure 3:**
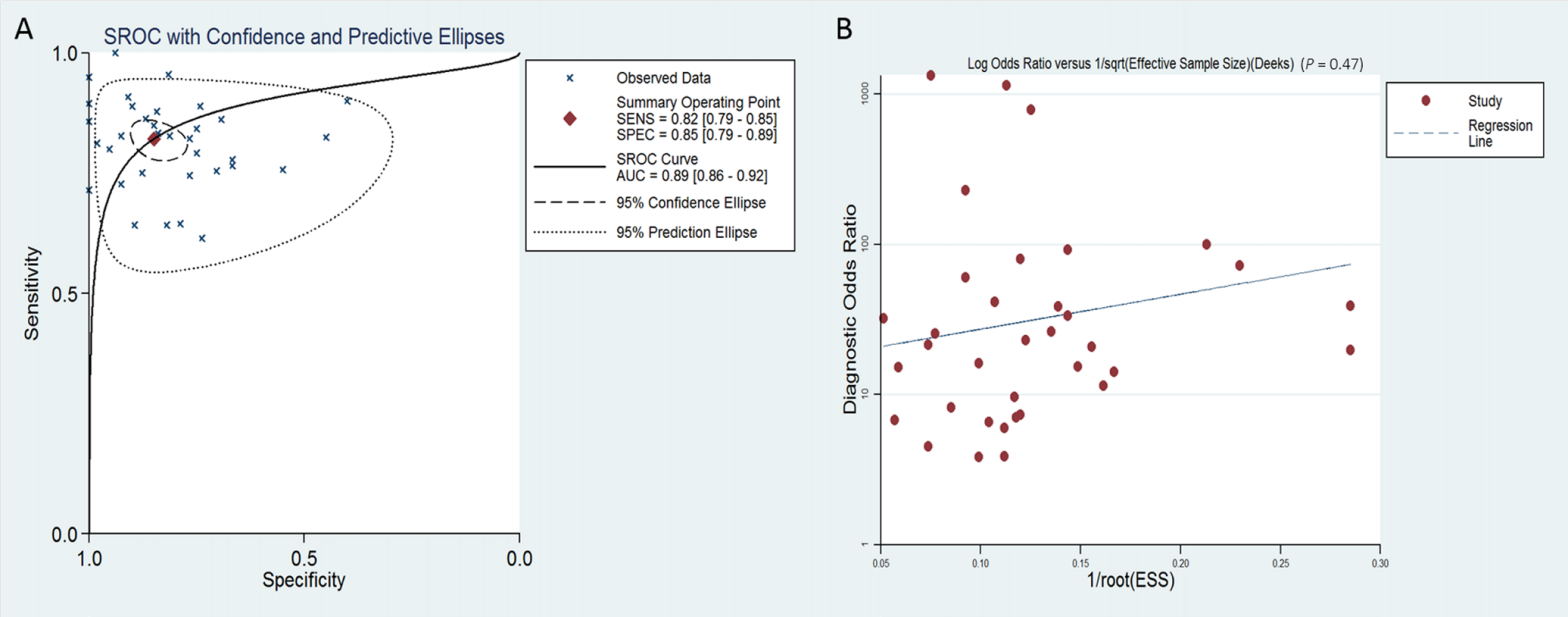
SROC curve with confidence and prediction regions around mean operating sensitivity and specificity points (**A**) and Deeks’ test for assessing publication bias (**B**) for miRNA in the diagnosis of pancreatic cancer.

The overall pooled SEN and SPE for 36 studies were 82% (95% CI, 79–85%) and 85% (95% CI, 79–89%), respectively, for distinguishing patients with PaCa from healthy controls (Figure 2A and 2B). Meanwhile, significant heterogeneity was observed in SEN and SPE since *I^2^* for SEN was 76.34% (95% CI, 68.82–83.85), and *I^2^* for SPE was 82.75% (95% CI, 77.73–87.77). We therefore used the random-effect model for analysis. The PLR and NLR were 5.44 (95% CI, 3.85–7.68) and 0.21 (95% CI, 0.17–0.26), respectively (Supplementary Figure 1A and 1B), and the DOR was 29.95 (95% CI, 15.69–42.94) (Supplementary Figure 2). Figure 3A showed the summary receiver operating characteristic (SROC) curve, and the AUC of these 36 studies was 0.89 (95% CI, 0.86–0.92). Fagan's nomogram for likelihood ratios is shown in Supplementary Figure 3.

Next, pooled SEN, SPE, and AUC for 25 studies in which miRNA was measured in blood were 81% (95% CI, 77–85%), 84% (95% CI, 79–88%), and 0.89 (95% CI, 0.86–0.92), respectively; SEN, SPE, and AUC in 11 studies which measured miRNA in other body fluids (such as stool, bile, pancreatic cyst fluid, pancreatic juice, and salivary) were 85% (95% CI, 81–88%), 87% (95% CI, 70–95%), and 0.85 (95% CI, 0.82–0.88) respectively (Table 2).

**Table 2:** Summary estimates of subgroup analysis for miRNA in the diagnosis of pancreatic cancer

Subgroups	Numberof studies	Sensitivity(95% CI)	Specificity(95% CI)	PLR(95% CI)	NLR(95% CI)	DOR(95% CI)	AUC(95% CI)
Type of patients							
PDAC	16	0.87 [0.81, 0.91]	0.86 [0.78, 0.92]	6.38 [3.67, 11.10]	0.16 [0.11, 0.23]	40.95 [16.88, 99.35]	0.93 [0.90–0.95]
Pancreatic cancer	20	0.80 [0.76, 0.83]	0.83 [0.75, 0.89]	4.67 [3.06, 7.14]	0.25 [0.20, 0.31]	19.06 [10.61, 34.23]	0.86 [0.83–0.89]
Ethnicity							
Asian	21	0.79 [0.75, 0.83]	0.79 [0.71, 0.85]	3.74 [2.67, 5.23]	0.26 [0.21, 0.33]	14.27 [8.70, 23.40]	0.85 [0.82–0.88]
Caucasian	15	0.87 [0.81, 0.91]	0.91 [0.85, 0.95]	10.09 [5.50, 18.51]	0.15 [0.10, 0.21]	68.93 [28.36, 167.58]	0.94 [0.92–0.96]
MiRNA profiling							
Multiple miRNA	19	0.85 [0.80, 0.89]	0.89 [0.83, 0.92]	7.46 [4.82, 11.53]	0.17 [0.12, 0.23]	44.41 [22.07, 89.38]	0.93 [0.90–0.95]
Single miRNA	17	0.78 [0.74, 0.82]	0.79 [0.69, 0.86]	3.70 [2.48, 5.53]	0.28 [0.22, 0.34]	13.43 [7.74, 23.28]	0.84 [0.80–0.87]
Sample types							
Blood	25	0.81 [0.77, 0.85]	0.84 [0.79, 0.88]	5.10 [3.68, 7.08]	0.22 [0.17, 0.29]	22.88 [13.29, 39.38]	0.89 [0.86–0.92]
Not blood	11	0.85 [0.81, 0.88]	0.87 [0.70, 0.95]	6.54 [2.63, 16.27]	0.17 [0.13, 0.23]	37.57 [12.74, 110.77]	0.85 [0.82–0.88]

### Diagnostic efficacy of multiple miRNAs and a single miRNA in PaCa

**Figure 4:**
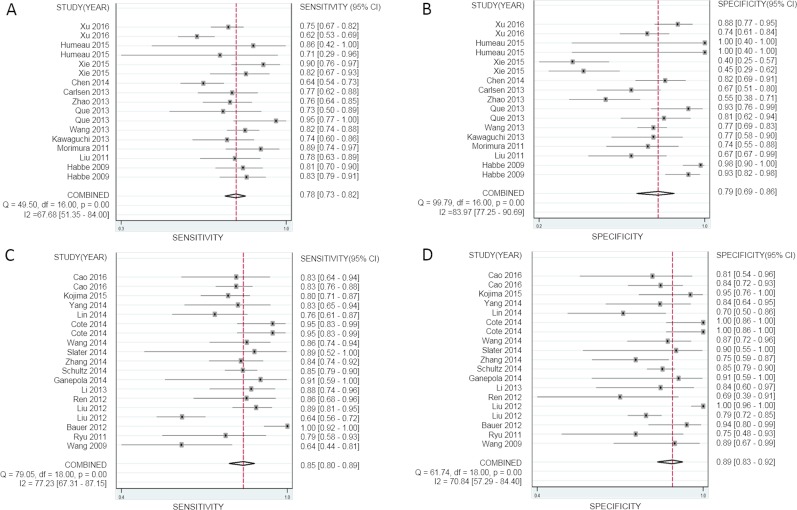
Forest plots of sensitivity and specificity for a single miRNA and multiple miRNAs in the diagnosis of pancreatic cancer (**A**, **B**) sensitivity and specificity of a single miRNA, (**C**, **D**) sensitivity and specificity of multiple miRNAs.

Evaluating the diagnostic accuracy of multiple miRNAs in the 19 studies, we found that the SEN was 85% (95% CI, 80–89%) (Figure 4C), the SPE was 89% (95% CI, 83–92%) (Figure 4D), and the AUC was 0.93 (95% CI, 0.90–0.95) (Supplementary Figure 4B); for the diagnostic accuracy of a single miRNA in the 17 studies, we found that the SEN was 78% (95% CI, 74–82%) (Figure 4A), the SPE was 79% (95% CI, 69–86%) (Figure 4B), and the AUC was 0.84 (95% CI, 0.80–0.87) (Supplementary Figure 4A), which showed significant divergences between multiple miRNAs and single miRNA, indicating that multiple miRNA profiling is more accurate in diagnosing PaCa.

### Ethnic subgroup analysis in pancreatic cancer based on circulating miRNA profiles

Subgroup analyses were also conducted and the pooled results are shown in Table 2. Different ethnic subgroup analysis revealed that in the 20 studies the accuracy of miRNAs to differentiate PaCa from controls, the SEN was 80% (95% CI, 76–83%), the SPE was 83% (95% CI, 75–89%), and the AUC was 0.86 (95% CI, 0.83–0.89). However, in the 16 studies for patients with PDAC, the SEN was 87% (95% CI, 81–91%), the SPE was 86% (95% CI, 78–92%), and the AUC was 0.93 (95% CI, 0.90–0.95), indicating a higher accuracy compared with applications on PaCa patients. Subgroup analysis in the 21 studies conducted in Asian populations, the SEN and SPE were 79% (95% CI, 75–83%) and 79% (95% CI, 71–85%), the AUC was 0.85 (95% CI, 0.82–0.88); for the 15 studies performed in Caucasian populations, these values were 87% (95% CI, 81–91%), 91% (95% CI, 85–95%) and 0.94 (95% CI, 0.92–0.96), which showed that there were significant divergences between Caucasian and Asian subgroups for circulating miRNA analysis.

## DISCUSSION

Due to increasing incidence, mortality and low survival rates of PaCa, screening is an urgent clinical challenge. However, accurate, affordable and repeatable detection methods are lacking. Even though miRNAs may have high diagnostic value [36], the clinical utility of miRNA for diagnosing pancreatic cancer remains disputable. There were more studies and participants included in this meta-analysis than Wan et al. [5] and Ding et al. [34]. In this study, we confirmed that miRNAs can be highly sensitive and specific diagnostic markers for PaCa. Higher accuracy was observed in multiple miRNAs profiling assays. We conducted this meta-analysis to evaluate the diagnostic accuracy of miRNA as a novel biomarker in diagnosing PaCa. A total of 36 studies in 29 articles conducted between 2009 and 2016 involving a total of 2225 PaCa patients and 1618 controls were involved in this meta-analysis. The meta-analysis revealed that the pooled SEN was 82% (95% CI, 79–85%) and the pooled SPE was 85% (95% CI, 79–89%).

AUC is widely used for evaluating the accuracy of diagnostic tests; numbers greater than 0.9, between 0.9 and 0.7, and 0.7 and 0.5 indicate high, moderate and low diagnostic values, respectively. We found that in our meta-analysis, the area under the SROC curve (AUC) was 0.89 (0.86–0.92), suggesting that miRNA has a high diagnostic value for pancreatic cancer. DOR, as an evaluation index of diagnostic tests, was used to determine the relationships between the chances of getting positive and negative results. Our results showed that the pooled DOR was 29.95 (95% CI, 15.69–42.94), indicating that the chance that a subject testing positive for a PaCa miRNA has pancreatic cancer is 29.95 times higher than for those testing negative, which is a higher DOR than that of the traditional markers in serum such as CA19-9 [37].

It is worth noting that multiple miRNA assays were more accurate in diagnosing PaCa than single miRNAs. In multiple miRNA assays, miR-21 [7, 11, 12, 15, 17–20, 23, 31], miR-155 [12, 13, 15, 18, 19, 31] and miR-210 [15, 18, 19, 21, 30] were used most frequently in the included studies. Moreover, we further found that the diagnostic accuracy of miRNAs to differentiate PDAC from controls (SEN:87%, SPE: 86%, AUC: 0.93) indicating a higher accuracy compared with applications on PaCa patients (SEN: 80%, SPE:83%, AUC: 0.86) (Table 2). We also performed a publication bias test and showed that there was no publication bias. We also analyzed the publication bias applying Deeks’ funnel plot asymmetry test. The *p*-value for the test was 0.47 for all miRNAs diagnosing PaCa (Figure 3B), 0.71 for a single miRNA diagnosing PaCa (Supplementary Figure 5A), and 0.50 for multiple miRNAs diagnosing PaCa (Supplementary Figure 5B), indicating that there was no publication bias. However, we also recognized that the following limitations of this meta-analysis should be considered in interpreting the results, such as heterogeneity among the 36 studies; differences in miRNA profiling methods; specimen resources; relevant studies that might have been missed, or have not yet been published online; no statistical data concerning African populations, etc.

In conclusion, our meta-analysis noted the practicability of miRNA for diagnosing PaCa, and demonstrated that multiple miRNAs had a relatively high diagnostic value for pancreatic cancer compared to single miRNA diagnosis. Future studies still need to confirm the accuracy of using multiple miRNAs as biomarkers for noninvasive screening and diagnosis of PaCa in clinical applications.

## MATERIALS AND METHODS

### Search strategy

We performed our meta-analysis on the basis of the manuals of the Preferred Reporting Items for Meta-analyses (PRISMA). We conducted a document search for studies analyzing the diagnostic value of circulating miRNAs in patients with PaCa using PubMed, Medline, Embase, Google Scholar and Chinese National Knowledge Infrastructure (CNKI) databases. We identified the studies with the key words: (“pancreatic cancer” or “pancreatic tumor” or “pancreatic carcinoma” or “pancreatic neoplasm” or “pancreatic ductal adenocarcinoma”) and (“microRNA” or “miRNA”) and (“diagnosis” or “ROC curve” or “sensitivity” or “specificity”) up to October 1, 2016. We also scanned the reference of reviews and conference summaries in the initial search to find any additional acceptable articles.

### Study selection

A set of criteria was proposed for study inclusion. To be included, studies had to meet the following criteria: 1) patients with pancreatic cancer, 2) evaluate the diagnostic value of circulating miRNAs in PaCa, and 3) a diagnostic four-fold contingency table that could be calculated or extracted from the articles. The following exclusion criteria were: studies that were commentaries, reviews, or duplicate publications, studies unrelated to diagnosing pancreatic cancer using miRNA, and studies without complete data comparison groups.

### Data extraction and quality assessment

Two of the authors independently extracted the following data from the full text of the selected articles: first author's name; year and country of publication; subjects’ ethnicity, sex, and age; total number of cases and controls; miRNAs studied; type of specimen used for miRNA testing; SEN, SPE, true-positive (TP), false-positive (FP), false-negative (FN), and true-negative (TN) values of tested miRNAs. The following data were extracted by two of the authors independently from the eligible studies: first author's name, publication year, subjects’ ethnicity, miRNAs studied, specimen, total number of cases and controls, mean age, SEN, SPE, TP, FP, FN, TN and information needed for quality assessment. Study quality was systematically evaluated according to QUADAS-2 guidelines [9].

### Statistical methods

Statistical analyses were accomplished using the Stata 12.0 software (Stata-Corp, College Station, TX, version 12.0) and RevMan5.3 (version 1.4) software. We extracted or calculated the number of TP, FP, FN, and TN from each study. A bivariate random effects regression model was used to calculate the pooled sensitivity (TP/[TP + FN]), specificity (TN/[TN + FP]), DOR (diagnostic odds ratio), PLR (positive likelihood ratio), and NLR (negative likelihood ratio). We determined the SEN and SPE in the study using a bivariate summary receiver operating characteristic (SROC) curve and calculated the AUCs and 95% confidence intervals [38]. Heterogeneity inspection was performed using Higgin's I-squared statistic [39], an *I*^2^ greater than 50% suggested heterogeneity in the studies. Subgroup analysis was applied to detect sources of heterogeneity. Deeks’ funnel plot asymmetry test was employed to assess publication bias.

## SUPPLEMENTARY MATERIALS FIGURES



## Abbreviations

AUC: the area under the SROC curve
CNKI: chinese national knowledge infrastructure
DOR: diagnostic odds ratio
FN: false negative
FP: false positive
GI: gastrointestinal
IHC: immunohistochemistry
NLR: negative likelihood ratio
PaCa: pancreatic cancer
PDAC: pancreatic ductal adenocarcinoma
PLR: positive likelihood ratio
PRISMA: preferred Reporting Items for Meta-analyses
qRT-PCR: quantitative real-time polymerase chain reaction
SEN: sensitivity
SPE: specificity
SROC: summary receiver operating characteristic
TN: true negative
TP: true positive
