# Supplementary material for: Clinically relevant circulating microRNA profiling studies in pancreatic cancer using meta-analysis
Source: Oncotarget. 2017 Feb 7;8(14):22616–24. doi: 10.18632/oncotarget.15148 (PMC5410249; doi:10.18632/oncotarget.15148)
Supplement: Supplementary file 1 [file oncotarget-08-22616-s001.pdf]

# Clinically relevant circulating microRNA profiling studies in pancreatic cancer using meta-analysis

## Supplementary Materials

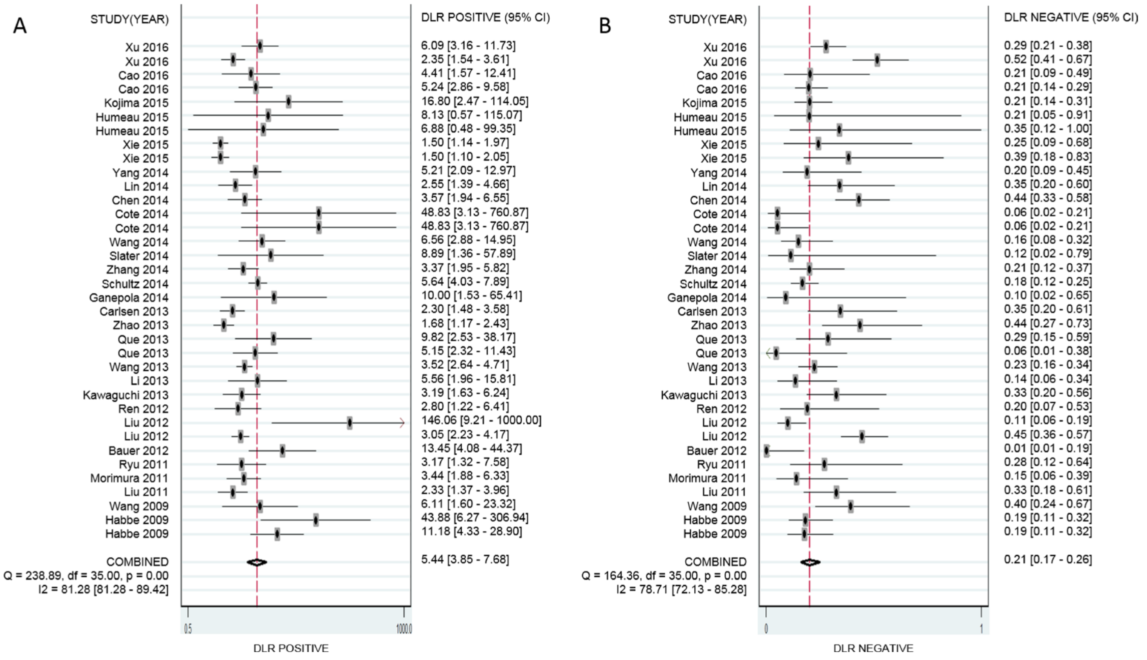


**Supplementary Figure 1:** Forest plots of PLR (**A**) and NLR (**B**) for miRNA in the diagnosis of pancreatic cancer.

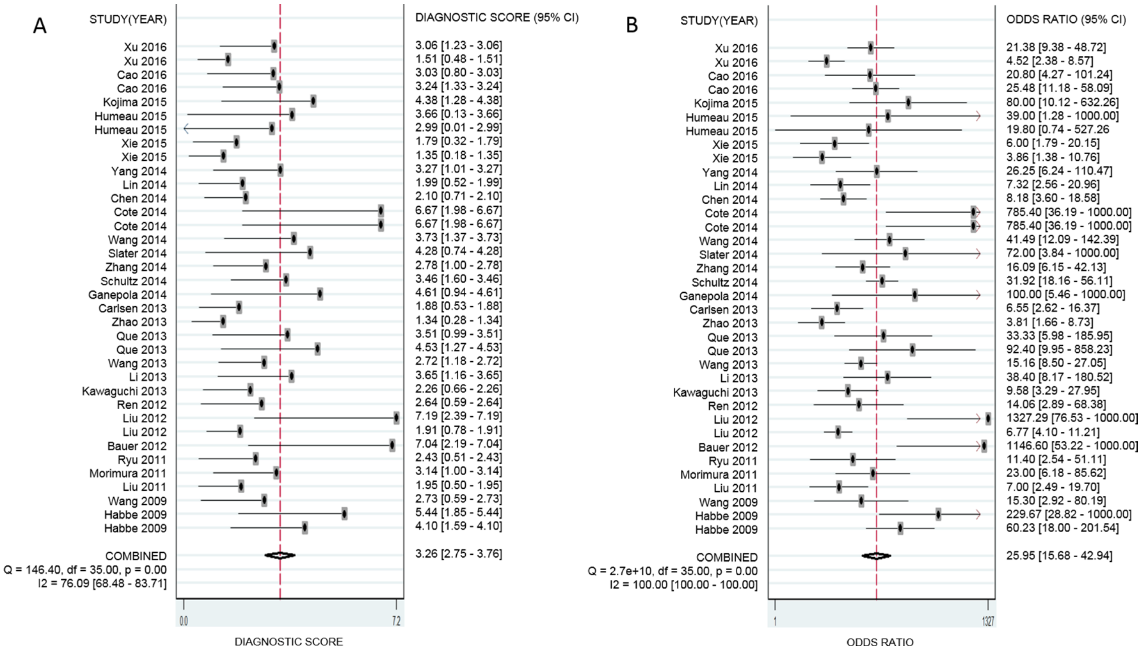


**Supplementary Figure 2:** Forest plots of diagnostic score (**A**) and odds ratios (**B**) for miRNAs in the diagnosis of pancreatic cancer.

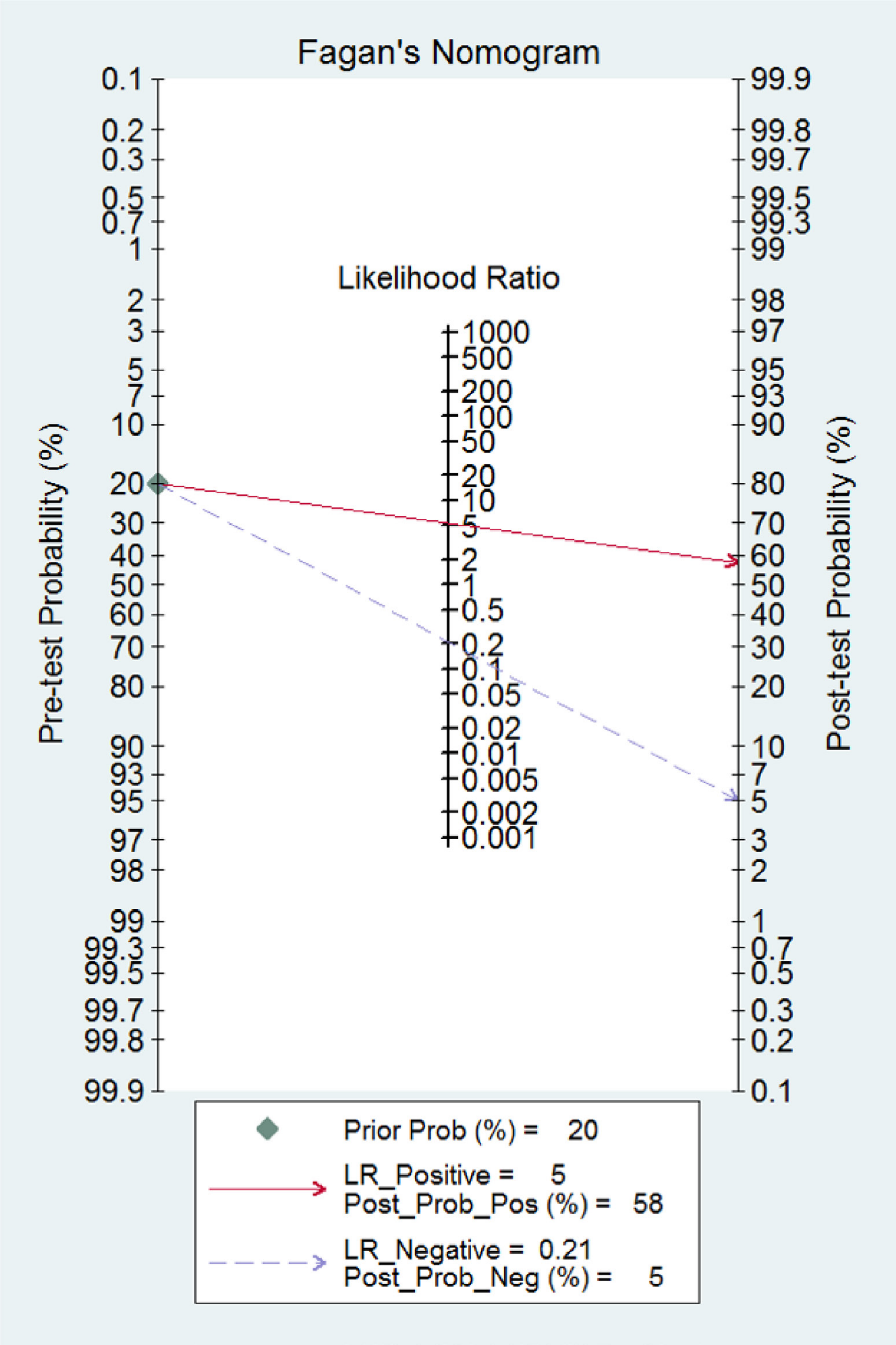


**Supplementary Figure 3: Fagan's nomogram for likelihood ratios.**

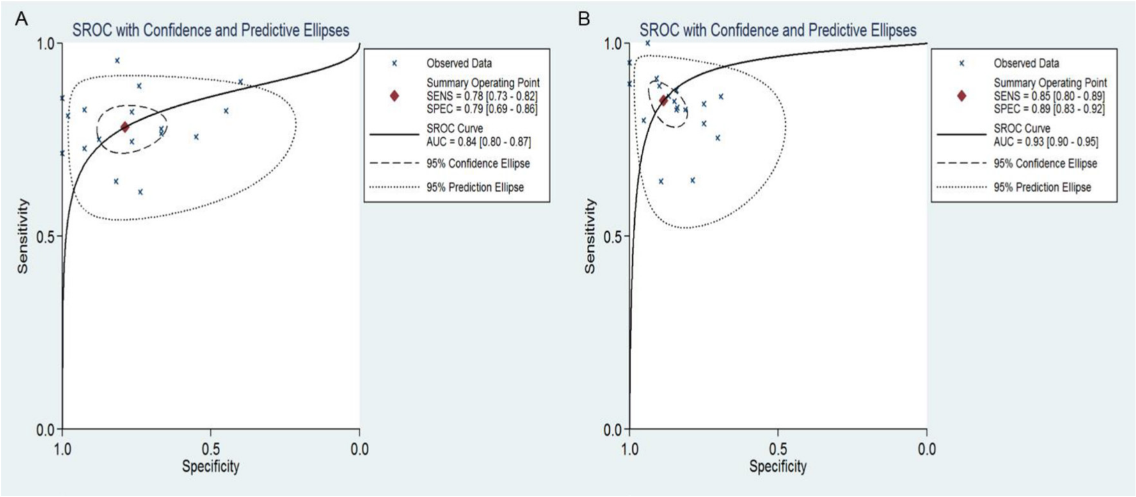


**Supplementary Figure 4:** SROC curve for single miRNA (**A**) and multiple miRNAs (**B**) in the diagnosis of pancreatic cancer.

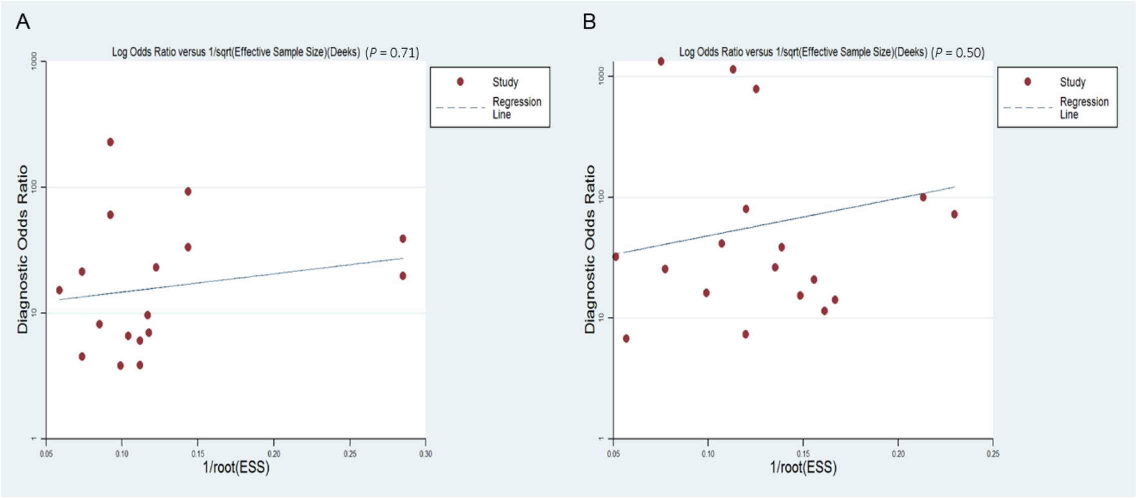


**Supplementary Figure 5:** Deeks' test for assessing publication bias of a single miRNA (**A**) and multiple miRNAs (**B**).
